# Analysis of nitrogen oxides (NOx) in the exhaled breath condensate (EBC) of subjects with asthma as a complement to exhaled nitric oxide (FeNO) measurements: a cross-sectional study

**DOI:** 10.1186/1756-0500-4-202

**Published:** 2011-06-16

**Authors:** Nathalie Chérot-Kornobis, Sébastien Hulo, Jean-Louis Edmé, Virginie de Broucker, Régis Matran, Annie Sobaszek

**Affiliations:** 1 Univ Lille Nord de France, F-59 000 Lille, France; 2 UDSL, EA 4483, F-59 000 Lille, France; 3 CHU Lille, F-59 000 Lille, France

## Abstract

**Background:**

The study of pulmonary biomarkers with noninvasive methods, such as the analysis of exhaled breath condensate (EBC), provides a useful approach to the pathophysiology of asthma. Although many recent publications have applied such methods, numerous methodological pitfalls remain. The first stage of our study consisted of validating methods for the collection, storage and analysis of EBC; we next sought to clarify the utility of analysing nitrogen oxides (NOx) in the EBC of asthmatics, as a complement to measuring exhaled nitric oxide (FeNO).

**Methods:**

This hospital-based cross-sectional study included 23 controls matched with 23 asthmatics. EBC and FeNO were performed and respiratory function measured. Intra-assay and intra-subject reproducibility were assessed for the analysis of NOx in the EBC of 10 healthy subjects.

**Results:**

The intraclass correlation coefficient (ICC) was excellent for intra-assay reproducibility and was moderate for intra-subject reproducibility (Fermanian's classification). NOx was significantly higher in asthmatics (geometric mean [IQR] 14.4 μM [10.4 - 19.7] vs controls 9.9 μM [7.5 - 15.0]), as was FeNO (29.9 ppb [17.9 - 52.4] vs controls 9.6 ppb [8.4 - 14.2]). FeNO also increased significantly with asthma severity.

**Conclusions:**

We validated the procedures for NOx analysis in EBC and confirmed the need for assays of other biomarkers to further our knowledge of the pathophysiologic processes of asthma and improve its treatment and control.

## Background

Inflammatory and oxidative changes in lungs are early indicators of the pathophysiology of many respiratory diseases [1-4]. Over the past decade, investigations of exhaled breath have multiplied, through the study of various compounds, both volatile, such as NO (FeNO), and non-volatile, especially nitrogen oxides (NOx). Understanding asthma requires markers of disease severity and control that can help to predict the risk factors for exacerbation [5,6]. Clinical and spirometric data are used in standardised questionnaires for follow-up and control. A more precise approach to the pathophysiology of asthma through the study of pulmonary biomarkers is interesting for early or even subclinical asthma diagnoses [4].

The study of FeNO is well standardised today; this compound is thought to reflect inflammatory conditions in the tissue (airway wall or alveolar compartments) [7-10]. Nonetheless, this biomarker does not provide any specific help in understanding the pathophysiological processes in asthma [11,12]: that is, it does not provide information on the phenomena of oxidative stress that play an important role in its pathogenesis. Moreover, FeNO is difficult to measure in young children [2]. On the other hand, the study of nonvolatile compounds through exhaled breath condensates (EBC) appears promising in this regard. This noninvasive method is both simple and reproducible, making it possible to explore the more specific factors of both inflammation and oxidative stress, through the study of NOx in EBC [13-15]. Despite the wide interest in this method, many methodological pitfalls remain, including the choice of systems for collection, storage and analysis [3,11]. The high intra assay variability of biomarker measurements is an important factor that explains its limited use in daily clinical practice [10,16]. The necessity for standardisation has been the subject of numerous publications over the past five years [11].

The first stage of our study consisted of validating the methods for the collection, storage, and analysis of EBC; we then used these rigorous methods to clarify the utility of analysis of NOx in the EBC of subjects with asthma, as a complement to FeNO measurements. We compared these two biomarkers in a population of patients with asthma and of controls matched for age and smoking habits.

## Methods

### Study population

This cross-sectional single-center study included 46 volunteer subjects over 18 years of age: 23 healthy non-atopic controls and 23 subjects with asthma, matched for age and smoking habits. Subjects were matched for age in 5-year age groups and for smoking habits by status.

The inclusion criterion for controls was normal lung function. Subjects were excluded if they were: pregnant, had smoked or drunk coffee within the past 4 hours, had respiratory disorders, had had recent infections (< 3 months) or potential environmental or occupational risk factors. The inclusion criterion for all subjects with asthma was the presence of moderate (GINA3) or severe (GINA4) persistent asthma [5]. Patients treated by inhaled corticosteroids and bronchodilators were accepted. The exclusion criteria were the same as for controls. The Institutional Review Board of Lille University Hospital approved this study.

### Questionnaire

The clinical evaluation was based on a standardised BMRC questionnaire [17].

### Lung function

The lung function tests were conducted according to the 2005 guidelines of the European Respiratory Society [18,19] and included both slow and forced spirometry, as well as single-breath CO transfer (TLCO). Slow vital capacity (SVC), maximum forced expiratory volume in a second (FEV1), total lung capacity (TLC), FEV1/SVC ratio and single-breath CO transfer (DLCO) were examined.

### FeNO measurement

FeNO levels were measured with a chemiluminescence NO analyzer (NOx 8000, SERES Aix en Provence, France). The examinations were conducted according to the ATS guidelines [20]]. Subjects were seated and noseclips were not used. After deep inspiration of air without NO (NO-free), NO concentrations were measured during controlled expiration, at the first stable FeNO plateau for at least three seconds (FeNO variations < 10% or 1 ppb). FeNO was measured twice for each of the four expiratory flows: 25, 50, 100, and 150 mL/s. We used for our study the mean FeNO of two measurements at 50 mL/s (FeNO_50_) and alveolar and bronchial NO production (respectively CalvNO and J'awNO) determined from Tsoukias's model [21] if the R^2 ^of the model was greater than 0.64 [22]. We studied the reproducibility of exhaled NO for 31 controls. The intraclass correlation coefficients for the four flows exceeded 0.98 [23].

### Collection of EBC

EBC were collected by an Ecoscreen device (Jaeger™, Würzburg, Germany), previously described [24]. An electronic spirometer (Ecovent Viasys, Hechberg, Germany) attached to the expiratory circuit allowed us to monitor the subject's ventilation. Collection was completed when the total expired volume reached 200 liters.

### Analysis of EBC

The total nitrites (NOx) were assayed by the Griess method (Griess Reagent Kit, Invitrogen, Cergy Pontoise, France) with spectrophotometric detection after reduction of nitrates. The concentration measured represents the sum of the nitrites and nitrates initially present in the EBC. We used an enzymatic technique in tubes, with nitrate reductase (Sigma, St. Quentin Fallavier, France) for the reduction stage. The limit of detection (LOD) was 2 μM. The detection rate in our population was 100% for the subjects with asthma and the controls. After collection, the condensates were immediately divided into aliquots and stored at -80°C [25].

In a preliminary study to examine long-term storage of the biomarkers in the condensates, we assayed the NOx present in the aliquots from a single sample, stored at -80°C and thawed at 1 day, 7 days, 1 month and 2 months after sampling. The short-term stability after thawing and storage of the aliquots at 4°C was also assessed.

The reproducibility of the assays for biomarkers in the EBC was tested in duplicate for 10 healthy controls two days in a row, so that we could study the reproducibility between assays and intra-individual reproducibility on two consecutive days.

### Statistical analysis

The statistical analyses were performed with SAS software (Cary NC). The intra-assay and intra-subject reproducibility were assessed by the coefficient of variation (CV) and the intraclass correlation coefficient (ICC). Excellent agreement for the ICC was defined as ranging from 1.00 to 0.91, good from 0.90 to 0.71, moderate from 0.70 to 0.51, slight from 0.50 to 0.31, and poor from 0.30 to 0.00 (Fermanian 1984). We also used the method recommended by Bland and Altman [26] to determine the bias and coefficient of repeatability between two measurements. Results were presented as geometric means with their interquartile ranges [IQR]. Descriptive statistics, with tests of normality (Shapiro-Wilk test) for the FeNO and NOx values in EBC, showed lognormal distributions. Statistical tests were performed after logarithmic transformation of the data. When the values were below the LOD, they were assigned a value of 0.5 LOD. The association between FeNO and NOx in EBC was determined with the Spearman correlation. Analysis of variance (ANOVA) was used to compare patients and controls. Analyses of more than two groups used Tukey's multiple comparison procedure.

## Results

### Stability and reproducibility

We found good long-term stability (93%) for the NOx measurements. This demonstrates very low loss during storage relative to the values obtained immediately after sampling. The NOx assays performed on three consecutive days (short-term stability) remained consistently stable (98%). These results are similar to these achieved by other teams [26,27].

The intra-assay and intra-subject reproducibility of the NOx assay in the EBC of 10 controls were assessed. Table 1 summarises the results of this analysis. The ICC for intra-assay reproducibility was excellent, according to Fermanian's classification. The ICC for intra-individual reproducibility over two consecutive days was moderate. Bland and Altman's method applied to the difference in the data obtained in these two studies of reproducibility shows that all the assays are within the limits of agreement.

**Table 1:** Intra-assay and intra-individual reproducibility of NOx analysis in EBC

	ICC^1^	CV^2^	Coef of Repeatability^3^	Bias^3^
Intra-assay	0.98	4.9% (4.7)	0.28	1.0 μM
Intra-individual	0.58	21.8% (14.3)	1.9	1.8 μM

1 Intraclass correlation coefficient

2 Coefficient of variation: mean (SD)

3 Bland and Altman test

### Subjects with asthma vs controls

Table 2 presents the general characteristics of the subjects with asthma and the matched controls. The results of the lung function tests show lower values in patients. The conditions of EBC collection were similar for the subjects with asthma and the controls, for both the duration and volume of collection.

**Table 2:** Patient's characteristics

	Asthma (n = 23)	Controls (n = 23)	Statistics^1^
Age (years) ^a^	50.0 [30.0 - 56.0]	46 [28.0 - 51.0]	0.534
Height (meter) ^a^	1.65 [1.62 - 1.75]	1.68 [1.62 - 1.75]	0.767
Weight (kg) ^a^	73.0 [63.0 - 84.0]	70.0 [61.0 - 75.0]	0.347
BMI (kg/m2) ^a^	26.5 [22.5 - 27.7]	23.4 [21.7 - 26.1]	0.173
Male ^b^	12 (52.2)	7 (30.4)	0.134
Smoking ^b^			1.0
Ex-smoker	4 (17.4)	4 (17.4)	
Smoker	6 (26.1)	6 (26.1)	
Non-smoker	13 (56.5)	13 (56.5)	
TLC (obs/pred) ^a,c^	106.4 [100.6 - 116.0]	108.6 [100.2 - 112.6]	0.859
SVC (obs/pred) ^a,c^	90.5 [81.0 - 95.4]	106.6 [100.4 - 119.2]	**< 0.001**
FEV_1 _(obs/pred) ^a,c^	56.5 [50.3 - 73.7]	107.1 [100.8 - 115.6]	**< 0.001**
FEV_1_/SVC (obs/pred) ^a,c^	69.4 [57.2 - 77.9]	102.8 [96.7 - 105.9]	**< 0.001**
MMEF (obs/pred) ^a,c^	23.4 [14.5 - 38.5]	95.7 [79.2 - 107.6]	**< 0.001**
TLCO (obs/pred) ^a,c^	78.5 [68.5 - 94.0]	90.0 [81.0 - 103.5]	0.102
			
Total expired volume (L) ^a^	204 [200 - 250]	205 [200 - 225]	0.629^2^
Total volume of EBC (mL) ^a^	4.00 [3.50 - 5.00]	4.00 [3.75 - 4.50]	0.787^2^
Time (min) ^a^	20 [16 - 25]	20 [18 - 24]	0.911^2^

BMI: body mass index, TLC: total lung capacity, SVC: slow vital capacity, FEV_1_: forced expiratory volume in 1s, MMEF: Maximum mid-expiratory flow, TLCO: carbon monoxide gas transfer

^a ^data expressed as median [IQR]

^b ^data expressed as n (%)

^c ^data expressed as % of predicted values

^1 ^p(Chi-2)

^2 ^ANOVA on the ranks

Table 3 summarises the biomarker levels in exhaled air. There was a statistically significant increase in the FeNO_50 _concentrations (p < 0.001), CalvNO (p = 0.002), J'awNO (p = 0.001) and NOx (p = 0.046) in the EBC of subjects with asthma compared with the controls. When we took into account the severity of asthma, this increase persisted for FeNO50 (p < 0.001), alveolar NO (p = 0.003) and bronchial NO (p = 0.005) (Table 4).

**Table 3:** Result of biomarkers in exhaled air

	Asthma (n = 23)	Controls (n = 23)	Statistics^b^
FeNO_50 _(ppb) ^a^	29.9 [17.9 - 52.4]	9.6 [8.4 - 14.2]	**< 0.001**
CalvNO (ppb)^a^	3.7 [2.7 - 6.3]	1.7 [1.1 - 2.8]	**0.002**
J'awNO (nL/min)^a^	80.6 [56.7 - 116.9]	30.9 [25.7 - 46.3]	**0.001**
NOx (μM) ^a^	14.4 [10.4 - 19.7]	9.9 [7.5 - 15.0]	**0.046**

FeNO_50_: Fractional exhaled nitric oxide at 50 mL/s, CalvNO: Alveolar NO concentration, J'awNO: Bronchial NO concentration, NOx: Nitrogen oxides

a data expressed as geometric mean [IQR]

b ANOVA

**Table 4:** Result of biomarkers in exhaled air, taking asthma severity into account

	GINA4 (n = 12)	GINA3 (n = 11)	Controls (n = 23)	Statistics^1^
FeNO_50 _^a^	32.6 [16.8 - 70.9]	27.6 [18.1 - 42.3]	9.6 [8.4 - 14.2]	**< 0.001 ^§,£^**
CalvNO (ppb) ^a^	2.8 [2.0 - 2.8]	4.4 [2.9 - 6.6]	1.7 [1.1 - 2.8]	**0.003 ^§^**
J'awNO (nL/min) ^a^	79.0 [44.3 - 175.4]	81.6 [58.5 - 114.4]	30.9 [25.7 - 46.3]	**0.005 ^§,£^**
NOx (μM) ^a^	12.5 [9.0 - 19.5]	16.9 [14.0 - 19.7]	9.9 [7.5 - 15.0]	0.074

FeNO_50_: Fractional exhaled nitric oxide at 50mL/s, CalvNO: Alveolar NO concentration, J'awNO: Bronchial NO concentration, NOx: Oxides of nitrogen

^a ^data expressed as geometric mean [IQR]

^1 ^ANOVA

^§ ^Significant increase GINA 3 vs controls

^£ ^Significant increase GINA 4 vs controls

We tested for correlations between the NOx in the EBC and exhaled NO but found no significant relationships.

## Discussion

This study validated the methodology of analysis of NOx in EBC and demonstrated satisfactory intra-assay and intra-individual reproducibility and consistent stability. The comparison of FeNO and NOx in EBC showed substantially higher concentrations in asthma patients. Moreover, the exhaled NO concentration increased when we took the severity of the asthma into account.

The study of exhaled NO is currently the most commonly used technique for which standardisation has been demonstrated to be compatible with clinical use [3]. Interest in EBC continues to increase because its safety and noninvasiveness make it a method of choice for studying the pathophysiologic mechanisms of lung diseases. Nonetheless, its development as a research tool remains slow because of a lack of standardisation and reference values for the biomarkers studied [3,27]. The standardisation that we sought to develop for the collection of condensates and the assay of biomarkers was thus an essential stage in advancing the use of EBC analysis for clinical and research purposes.

Our results showed an excellent level of standardisation for collection (Table 2) with a volume of 4 mL of EBC for a total expiratory volume set at 200 L. The decision to standardise according to expired volume rather than time thus appeared to be correct, as our results showed that the volumes of condensates exhibited low variability, both within and between control and patient groups.

The detection rate of NOx in the crude EBC was excellent. In a preceding study [24] we showed the importance of the choice of coating agent used for the various components of the EBC collection apparatus, as others have suggested [28-30], for optimising biomarker detection. Accordingly, the assays were performed on crude condensates thus avoiding sample handling and loss of biomarkers during lyophilisation and resolubilisation. These efforts provided very satisfactory detection, greater than 95%, for the NOx.

We also studied intra-assay and intra-individual reproducibility. The comparison of our results with those of other studies nonetheless remained difficult because these studies used different modes of sample collection and different assay techniques with different levels of reproducibility [11]. That is, most studies have examined intra-assay reproducibility [13,31,32]; reproducibility from day to day has been studied much more rarely, and intra-individual reproducibility on two consecutive days still less often [31,33]. Only a few other studies have, like ours, simultaneously examined the intra-assay and intra-subject reproducibility of the biomarkers. Our results for NOx in EBC (coefficients of variation (CVs) = 4.7%) were similar to those of other studies [13,31] who found mean CVs of 3.11% and 4.9% respectively, but better than those from an older study [32] which reported an ICC of 0.71 in the nitrite assay (ICC in this study = 0.98 for NOx).

The intra-subject reproducibility for NOx appeared similar to that found by Chladkova *et al. *[33] but was better than that reported by Chow *et al. *[31].

Relatively few previous studies have explored the conservation of markers in the condensates. Our results, better than those found by either Chladkova *et al. *[33] or Vogelberg *et al. *[34], showed that NOx stability was good in both the short and long term storage scenarios studied here.

It is not easy to compare our NOx levels with those in the literature because each team has assayed different forms of NOx (nitrites, nitrites/nitrates, total nitrites) by different methods (chemiluminescence, colourimetrics, fluorescence or ionex HPLC) [11]. Comparison with other studies is much easier for FeNO, which has the best standardisation [7-10]. Our mean levels in controls (9.6 ppb) were similar to those reported by other teams [13,35].

The FeNO study made it possible to discriminate between subjects with asthma and controls, but also between subjects with asthma of different degrees of severity and controls. Nonetheless, the hypothesis was that NOx accurately reflects oxidation in the EBC while FeNO is an indirect indicator of inflammation. That is, it is the product of arginine transformation by NO synthase and appears to be involved in the regulation of inflammation in pulmonary diseases. This unstable product reacts in aqueous solution with oxygen or the radical species formed by oxidative stress, such as the superoxide anion, to form, among other molecules, the relatively stable nitrogen oxides (NOx), including nitrites (NO_2_^-^) and nitrates (NO_3_^-^) [14,15]. This production increases in situations of oxidative stress. This is the reason behind our choosing to study this biomarker of oxidative stress, an important phenomenon to consider with asthma, and one that simultaneously but indirectly reflects inflammation. NOx production can vary according to the specific lung disease [13,14], the treatment administered [36] and smoking habits [37]. The impact of age on FeNO has been studied [38] but its impact on NOx production has been less studied [39]. Hence, we took care to match subjects with asthma and their controls for age and smoking status.

The concentrations of FeNO and of NOx in the EBC both differed significantly between subjects with asthma and controls. On the other hand, we did not find a significant difference according to the severity of asthma, although a trend was nevertheless evident (Table 4). Other authors have found significant differences in FeNO levels in patients with mild compared with severe asthma, but observed findings similar to ours for moderate and severe asthma, the categories compared in our study design [14,40]. The absence of a correlation in our study between NOx in the EBC and exhaled, alveolar, and bronchial NO may be explained by the use of different NO transformation pathways. The formation of peroxynitrite, which interacts with some amino acids, has harmful consequences on the properties of some proteins, which may explain the aggravation and even the lack of response to some treatment. Accordingly, assays of NOx alone in our study did not appear sufficient, and the exploration of this other pathway of NO transformation by the peroxynitrite assay might be useful in explaining the pathophysiology of asthma aggravation.

## Conclusions

The results of our study confirmed the pertinence of the study of FeNO in subjects with asthma. The NOx assay in EBC remained insufficient to provide supplementary information but may be interesting in populations where FeNO cannot be studied, as in young children [2]. The study of exhaled breath condensates therefore remained seductive as a clinical and research tool but the continuation of assays of other biomarkers remains essential to further our knowledge of the pathophysiologic processes of asthma and improve its treatment and control. We are working in this direction, continuing to assay NOx together with peroxynitrite and hydrogen peroxide (H_2_O_2_), in order to further our knowledge of other oxidation pathways and their role in the occurrence of asthma.

## Competing interests

The authors declare that they have no competing interests.

## Authors' contributions

NC, JL., VD, SH and AS participated in designing and performing the research; NC and JLE verified and analysed the data; NK wrote the article and all authors have verified and approved the final version of the manuscript.
